# (+)-{1,2-Bis[(2*R*,5*R*)-2,5-dimethyl­phospho­lan-1-yl]ethane-κ^2^ *P*,*P*′}(η^4^-cyclo­octa-1,5-diene)rhodium(I) tetra­fluorido­borate

**DOI:** 10.1107/S1600536810019859

**Published:** 2010-05-29

**Authors:** Stefan Schulz, Hans-Joachim Drexler, Detlef Heller

**Affiliations:** a Leibniz-Institut für Katalyse e. V. an der Universität Rostock, Albert-Einstein-Strasse 29a, 18059 Rostock, Germany

## Abstract

The title compound, [Rh(C_8_H_12_)(C_14_H_28_P_2_)]BF_4_, exhibits a rhodium(I) complex cation with a bidentate bis­phosphine ligand and a bidentate η^2^,η^2^-coordinated cyclo­octa-1,5-diene. Together the ligands create a slightly distorted square-planar cordination environment for the Rh(I) atom. There are three mol­ecules in the asymmetric unit and intra­molecular P—Rh—P bite angles of 82.78 (5), 82.97 (6) and 83.09 (5)° are observed. The dihedral angles between the P—Rh—P and the *X*—Rh—*X* planes (*X* is the centroid of a double bond) are 14.7 (1), 14.8 (1) and 15.3 (1)°. The structure exhibits disorder of one cyclo­octa­diene ligand as well as one BF_4_ anion.

## Related literature

For general synthetic procedures for cationic rhodium bis­phosphine diolefin complexes, see: Schrock & Osborn (1971 ▶); Fennis *et al.* (1990 ▶); Fernandez *et al.* (2000 ▶). For a discussion on the structures of cationic rhodium bis­phosphine diolefin complexes in general, see: Drexler *et al.* (2004 ▶) and for the different stereoisomers of [Rh(Me-BPE)COD]BF_4_, see: Fox & McCague (2005 ▶). For the structures of related complexes, see: Burk *et al.* (1990 ▶); Burk (1991 ▶); Drexler *et al.* (2004 ▶); Burk *et al.* (1993 ▶). For applications of related ligands in catalytic reactions, see: Axtell *et al.* (2005 ▶); Burk *et al.* (1995 ▶); Heller *et al.* (2002 ▶); Schäffner *et al.* (2008 ▶).
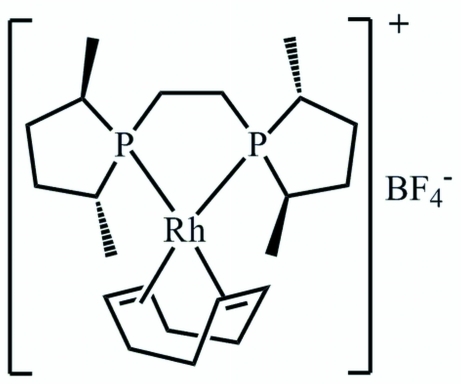


## Experimental

### 

#### Crystal data

[Rh(C_8_H_12_)(C_14_H_28_P_2_)]BF_4_
*M* *_r_* = 556.20
Orthorhombic, 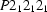

*a* = 10.224 (2) Å
*b* = 14.771 (3) Å
*c* = 50.158 (10) Å
*V* = 7575 (3) Å^3^
*Z* = 12
Mo *K*α radiation
μ = 0.84 mm^−1^
*T* = 200 K
0.20 × 0.13 × 0.10 mm

#### Data collection

Stoe IPDS 2 diffractometer
Absorption correction: numerical (*X-SHAPE* and *X-RED32*; Stoe & Cie, 2005 ▶) *T*_min_ = 0.853, *T*_max_ = 0.945
67186 measured reflections
13252 independent reflections
9566 reflections with *I* > 2σ(*I*)
*R*_int_ = 0.055

#### Refinement

*R*[*F* ^2^ > 2σ(*F* ^2^)] = 0.032
*wR*(*F* ^2^) = 0.046
*S* = 0.87
13252 reflections
804 parameters
77 restraints
H-atom parameters constrained
Δρ_max_ = 0.37 e Å^−3^
Δρ_min_ = −0.28 e Å^−3^
Absolute structure: Flack (1983 ▶), 5822 Friedel pairs
Flack parameter: −0.03 (2)

### 

Data collection: *X-AREA* (Stoe & Cie, 2005 ▶); cell refinement: *X-AREA*; data reduction: *X-RED32* (Stoe & Cie, 2005 ▶); program(s) used to solve structure: *SHELXS97* (Sheldrick, 2008 ▶); program(s) used to refine structure: *SHELXL97* (Sheldrick, 2008 ▶); molecular graphics: *SHELXTL* (Sheldrick, 2008 ▶); software used to prepare material for publication: *SHELXTL*.

## Supplementary Material

Crystal structure: contains datablocks I, global. DOI: 10.1107/S1600536810019859/im2198sup1.cif

Structure factors: contains datablocks I. DOI: 10.1107/S1600536810019859/im2198Isup2.hkl

Additional supplementary materials:  crystallographic information; 3D view; checkCIF report

## Figures and Tables

**Fig. 1.**
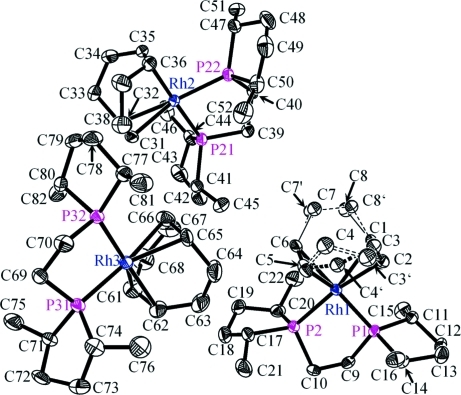
Perspective view and numbering scheme of the three cations [Rh((R,R)-Me-BPE)COD]+ in the asymmetric unit (30% probability displacement ellipsoids). All hydrogens and anions have been omitted for clarity.

**Fig. 2.**
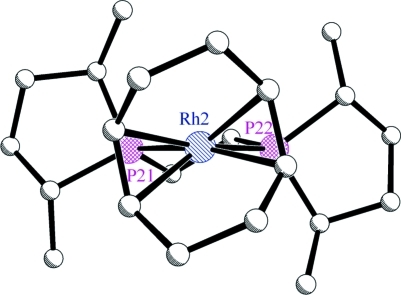
Front view, perspective diagram of one cation [Rh((R,R)-Me-BPE)COD]+ showing the anticlockwise twist of the cycloocta-1,5-diene ligand.

## supplementary crystallographic information

### Comment

In the course of our studies on structures of cationic rhodium bisphosphine diolefin complexes (Drexler *et al.*, 2004), we became interested in the chiral ligand family of 1,2-bis(2,5-dialkylphospholano)ethane, that build up five-membered ring chelates with rhodium (see: figure 1).

The title compound [Rh((*R*,*R*)-Me-BPE)COD)]BF_4 _can be easily synthesized by an cycloocta-1,5-diene (COD) exchange of [Rh(COD)_2_]BF_4_ with the chiral bisphosphine ligand (*R*,*R*)-Me-BPE (Schrock & Osborn, 1971) or an exchange of acetylacetonate (acac) by the bisphosphine in the presence of HBF_4_ starting from the precursor [Rh(acac)(COD)] (Fennis *et al.*, 1990).

Usually, the five-membered ring chelate is flexible and *λ*- or *δ*-conformers would be anticipated (Fernandez *et al.*, 2000). For the title compound the molecular structure of the three molecules in the asymmetric unit exclusively show *δ*-conformation of the bisphosphine backbones. COD ligands are *η*^2^,*η*^2^-coordinated and are orientated in an anticlockwise twist manner for all molecules (see: figure 2). This means that the double bonds of COD are not coordinated perpendicular to the P,Rh,*P* plane. The dihedral angles between the planes of P,Rh,*P* and of X,Rh,X (X = centroid of the double bond) are 14.8 (1)° (Rh1), 14.7 (1)° (Rh2) and 15.3 (1)° (Rh3). These dihedral angles are comparable to those in the corresponding Me-BPE complex [Rh((*R*,*R*)-Me-BPE)COD]SbF_6_ (19.4°) (Burk *et al.*, 1990; Drexler *et al.*, 2004) or for the Me-DuPhos complexes ([Rh((*S*,*S*)-Me-DuPhos)COD]BF_4_: 16.4°, Drexler *et al.*, 2004; [Rh((*S*,*S*)-Me-DuPhos)COD]SbF_6_: 17.8°, Drexler *et al.*, 2004; Burk *et al.*, 1993).

Intramolecular P—Rh—P angles of 82.78 (5)°, 82.97 (6)° and 83.09 (5)° were obtained. They are in the same range as those of corresponding complexes already described in the literature ([Rh((*R*,*R*)-Me-BPE)COD]SbF_6_: 83.25 (6)°, Burk *et al.*, 1990; [Rh((*R*,*R*)-Et-DuPhos)COD]BF_4_: 85.32 (6)°, Drexler *et al.*, 2001) exclusively for the complex [Rh((*R*,*R*)-*i*Pr-BPE)COD]SbF_6_ with an bite angle of 95.18 (9)° (Burk *et al.*, 1991).

### Experimental

By overlaying a solution of [Rh((*R*,*R*)-Me-BPE)COD]BF_4_ in dichloromethane with MTBE (methyl-*tert*-butylether) red-orange single crystals suitable for X-ray analysis are obtained. ^31^P NMR (CD_2_Cl_2_, 298 K, 122 MHz) [ppm]: 77.0 (d, *J*_P—Rh_ = 146.0 Hz).

### Refinement

All non-hydrogen atoms are refined anisotropically, except not fully occupied non-hydrogen atoms of one anion and one COD, which are disordered. Distance restraints were used to improve the geometry of the respective anions and COD ligands. All H atoms were placed in idealized positions with d(C—H) = 0.99 (CH_2_), 0.98 (CH_3_) and 1.0 Å (CH) and refined using a riding model with *U*_iso_(H) fixed at 1.5 *U*_eq_(C) for CH_3_ and 1.2 *U*_eq_(C) for CH_2_ and CH.

### Crystal data

**Table:**

[Rh(C_8_H_12_)(C_14_H_28_P_2_)]BF_4_	*F*(000) = 3456
*M**_r_* = 556.20	*D*_x_ = 1.463 Mg m^−^^3^
Orthorhombic, *P*2_1_2_1_2_1_	Mo *K*α radiation, λ = 0.71073 Å
Hall symbol: P 2ac 2ab	Cell parameters from 35629 reflections
*a* = 10.224 (2) Å	θ = 1.4–26.1°
*b* = 14.771 (3) Å	µ = 0.84 mm^−^^1^
*c* = 50.158 (10) Å	*T* = 200 K
*V* = 7575 (3) Å^3^	Part of rod, red
*Z* = 12	0.20 × 0.13 × 0.10 mm

### Data collection

**Table:**

Stoe IPDS 2 diffractometer	13252 independent reflections
Radiation source: fine-focus sealed tube	9566 reflections with *I* > 2σ(*I*)
graphite	*R*_int_ = 0.055
Detector resolution: 6.67 pixels mm^-1^	θ_max_ = 25.0°, θ_min_ = 1.4°
rotation method scans	*h* = −12→12
Absorption correction: numerical (*X-SHAPE* and *X-RED32*; Stoe & Cie, 2005)	*k* = −17→17
*T*_min_ = 0.853, *T*_max_ = 0.945	*l* = −59→59
67186 measured reflections	

### Refinement

**Table:**

Refinement on *F*^2^	Secondary atom site location: difference Fourier map
Least-squares matrix: full	Hydrogen site location: inferred from neighbouring sites
*R*[*F*^2^ > 2σ(*F*^2^)] = 0.032	H-atom parameters constrained
*wR*(*F*^2^) = 0.046	*w* = 1/[σ^2^(*F*_o_^2^) + (0.*P*)^2^] where *P* = (*F*_o_^2^ + 2*F*_c_^2^)/3
*S* = 0.87	(Δ/σ)_max_ = 0.005
13252 reflections	Δρ_max_ = 0.37 e Å^−^^3^
804 parameters	Δρ_min_ = −0.28 e Å^−^^3^
77 restraints	Absolute structure: Flack (1983), 5822 Friedel pairs
Primary atom site location: structure-invariant direct methods	Flack parameter: −0.03 (2)

### Special details

**Table:**

Geometry. All esds (except the esd in the dihedral angle between two l.s. planes) are estimated using the full covariance matrix. The cell esds are taken into account individually in the estimation of esds in distances, angles and torsion angles; correlations between esds in cell parameters are only used when they are defined by crystal symmetry. An approximate (isotropic) treatment of cell esds is used for estimating esds involving l.s. planes.
Refinement. Refinement of *F*^2^ against ALL reflections. The weighted *R*-factor wR and goodness of fit *S* are based on *F*^2^, conventional *R*-factors *R* are based on *F*, with *F* set to zero for negative *F*^2^. The threshold expression of *F*^2^ > σ(*F*^2^) is used only for calculating *R*-factors(gt) etc. and is not relevant to the choice of reflections for refinement. *R*-factors based on *F*^2^ are statistically about twice as large as those based on *F*, and *R*- factors based on ALL data will be even larger.

### Fractional atomic coordinates and isotropic or equivalent isotropic displacement parameters (Å^2^)

**Table:**

	*x*	*y*	*z*	*U*_iso_*/*U*_eq_	Occ. (<1)
Rh1	0.82592 (3)	0.50648 (3)	0.225528 (7)	0.03098 (9)	
Rh2	0.68473 (4)	0.17120 (3)	0.070150 (7)	0.03643 (10)	
Rh3	0.30533 (4)	0.65212 (3)	0.096621 (7)	0.03681 (10)	
P1	0.86072 (10)	0.52268 (9)	0.27014 (3)	0.0334 (3)	
P2	0.61906 (11)	0.47364 (9)	0.23907 (3)	0.0376 (3)	
P21	0.68588 (15)	0.12890 (9)	0.11366 (3)	0.0398 (3)	
P22	0.90416 (12)	0.17094 (11)	0.07562 (3)	0.0387 (4)	
P31	0.11683 (12)	0.73359 (10)	0.09291 (3)	0.0398 (4)	
P32	0.32525 (15)	0.68615 (9)	0.05279 (2)	0.0372 (3)	
C1	1.0259 (5)	0.4697 (4)	0.21284 (11)	0.0443 (15)	
H1A	1.0760	0.4408	0.2277	0.053*	0.50
H1B	1.0789	0.4382	0.2262	0.053*	0.50
C2	1.0220 (5)	0.5638 (4)	0.21560 (12)	0.0462 (16)	
H2A	1.0722	0.5886	0.2311	0.055*	0.50
H2B	1.0696	0.5869	0.2311	0.055*	0.50
C3	1.0234 (9)	0.6181 (9)	0.1923 (2)	0.045 (4)*	0.50
H3A	1.0627	0.5834	0.1775	0.053*	0.50
H3B	1.0783	0.6722	0.1955	0.053*	0.50
C4	0.8848 (8)	0.6481 (8)	0.1845 (2)	0.042 (3)*	0.50
H4A	0.8855	0.6706	0.1659	0.051*	0.50
H4B	0.8570	0.6984	0.1962	0.051*	0.50
C3'	0.9846 (14)	0.6392 (10)	0.1936 (3)	0.065 (5)*	0.50
H3C	0.9500	0.6932	0.2029	0.078*	0.50
H3D	1.0654	0.6576	0.1842	0.078*	0.50
C4'	0.8841 (10)	0.6086 (9)	0.1729 (2)	0.060 (4)*	0.50
H4C	0.8501	0.6615	0.1629	0.072*	0.50
H4D	0.9248	0.5662	0.1600	0.072*	0.50
C5	0.7798 (5)	0.5645 (5)	0.18707 (12)	0.0626 (19)	
H5A	0.6873	0.5863	0.1862	0.075*	0.50
H5B	0.6928	0.5915	0.1850	0.075*	0.50
C6	0.7889 (5)	0.4703 (5)	0.18289 (11)	0.063 (2)	
H6A	0.7077	0.4359	0.1783	0.076*	0.50
H6B	0.7014	0.4440	0.1810	0.076*	0.50
C7	0.9149 (9)	0.4465 (8)	0.1687 (2)	0.043 (3)*	0.50
H7A	0.9548	0.5015	0.1609	0.052*	0.50
H7B	0.8975	0.4028	0.1541	0.052*	0.50
C8	1.0077 (12)	0.4043 (9)	0.1895 (2)	0.048 (4)*	0.50
H8A	0.9707	0.3464	0.1960	0.058*	0.50
H8B	1.0935	0.3914	0.1811	0.058*	0.50
C7'	0.8969 (8)	0.3997 (8)	0.1755 (2)	0.040 (3)*	0.50
H7C	0.8748	0.3403	0.1835	0.048*	0.50
H7D	0.9002	0.3922	0.1559	0.048*	0.50
C8'	1.0314 (9)	0.4306 (9)	0.1857 (2)	0.037 (4)*	0.50
H8C	1.0917	0.3781	0.1858	0.045*	0.50
H8D	1.0674	0.4764	0.1733	0.045*	0.50
C9	0.7140 (4)	0.4873 (4)	0.28857 (9)	0.0419 (13)	
H9A	0.7141	0.4208	0.2910	0.050*	
H9B	0.7128	0.5161	0.3064	0.050*	
C10	0.5960 (4)	0.5158 (4)	0.27287 (10)	0.0389 (13)	
H10A	0.5879	0.5825	0.2728	0.047*	
H10B	0.5157	0.4897	0.2808	0.047*	
C11	1.0028 (5)	0.4737 (4)	0.28699 (11)	0.0421 (14)	
H11A	1.0766	0.4749	0.2739	0.051*	
C12	1.0378 (5)	0.5388 (4)	0.30895 (12)	0.0560 (17)	
H12A	1.1288	0.5288	0.3150	0.067*	
H12B	0.9784	0.5310	0.3244	0.067*	
C13	1.0227 (5)	0.6331 (4)	0.29706 (13)	0.0573 (17)	
H13A	1.0945	0.6450	0.2843	0.069*	
H13B	1.0268	0.6792	0.3114	0.069*	
C14	0.8908 (5)	0.6387 (3)	0.28275 (10)	0.0425 (13)	
H14A	0.8236	0.6502	0.2968	0.051*	
C15	0.9866 (6)	0.3764 (4)	0.29560 (13)	0.064 (2)	
H15A	1.0675	0.3553	0.3040	0.096*	
H15B	0.9673	0.3388	0.2800	0.096*	
H15C	0.9144	0.3720	0.3084	0.096*	
C16	0.8791 (5)	0.7152 (3)	0.26294 (12)	0.0620 (16)	
H16A	0.8968	0.7728	0.2719	0.093*	
H16B	0.7905	0.7161	0.2555	0.093*	
H16C	0.9425	0.7063	0.2485	0.093*	
C17	0.4699 (4)	0.4987 (5)	0.21980 (11)	0.0554 (16)	
H17A	0.4949	0.5009	0.2005	0.067*	
C18	0.3837 (5)	0.4154 (5)	0.22387 (14)	0.070 (2)	
H18A	0.3379	0.4193	0.2412	0.084*	
H18B	0.3172	0.4117	0.2096	0.084*	
C19	0.4703 (5)	0.3331 (5)	0.22324 (13)	0.0680 (18)	
H19A	0.5005	0.3215	0.2048	0.082*	
H19B	0.4212	0.2794	0.2295	0.082*	
C20	0.5874 (4)	0.3504 (4)	0.24144 (11)	0.0518 (15)	
H20A	0.5594	0.3372	0.2601	0.062*	
C21	0.4052 (6)	0.5867 (4)	0.22654 (14)	0.075 (2)	
H21A	0.3279	0.5949	0.2152	0.113*	
H21B	0.4667	0.6365	0.2235	0.113*	
H21C	0.3787	0.5863	0.2453	0.113*	
C22	0.7015 (6)	0.2899 (3)	0.23517 (13)	0.0694 (17)	
H22A	0.6753	0.2265	0.2373	0.104*	
H22B	0.7738	0.3034	0.2474	0.104*	
H22C	0.7299	0.3004	0.2168	0.104*	
C31	0.4901 (5)	0.2375 (4)	0.06897 (12)	0.0472 (15)	
H31A	0.4595	0.2587	0.0868	0.057*	
C32	0.4687 (4)	0.1448 (4)	0.06465 (12)	0.0498 (17)	
H32A	0.4255	0.1122	0.0797	0.060*	
C33	0.4467 (5)	0.1042 (5)	0.03785 (14)	0.083 (2)	
H33A	0.3955	0.1476	0.0270	0.099*	
H33B	0.3929	0.0490	0.0400	0.099*	
C34	0.5688 (6)	0.0794 (6)	0.02254 (15)	0.094 (3)	
H34A	0.5913	0.0159	0.0269	0.113*	
H34B	0.5480	0.0817	0.0033	0.113*	
C35	0.6846 (6)	0.1354 (4)	0.02726 (10)	0.0585 (16)	
H35A	0.7680	0.1070	0.0210	0.070*	
C36	0.6906 (7)	0.2298 (4)	0.02921 (10)	0.0593 (16)	
H36A	0.7772	0.2561	0.0241	0.071*	
C37	0.5777 (6)	0.2910 (5)	0.02433 (13)	0.090 (3)	
H37A	0.5202	0.2631	0.0107	0.109*	
H37B	0.6106	0.3488	0.0170	0.109*	
C38	0.4976 (7)	0.3108 (5)	0.04901 (12)	0.082 (2)	
H38A	0.5346	0.3651	0.0578	0.098*	
H38B	0.4075	0.3260	0.0433	0.098*	
C39	0.8513 (4)	0.1388 (4)	0.12698 (10)	0.0460 (15)	
H39A	0.8702	0.2025	0.1318	0.055*	
H39B	0.8609	0.1011	0.1432	0.055*	
C40	0.9448 (5)	0.1068 (4)	0.10544 (11)	0.0418 (15)	
H40A	0.9338	0.0411	0.1022	0.050*	
H40B	1.0366	0.1183	0.1108	0.050*	
C41	0.5772 (5)	0.1797 (4)	0.13869 (11)	0.0475 (15)	
H41A	0.4928	0.1937	0.1295	0.057*	
C42	0.5498 (6)	0.1038 (5)	0.15788 (13)	0.068 (2)	
H42A	0.6250	0.0948	0.1700	0.082*	
H42B	0.4714	0.1177	0.1687	0.082*	
C43	0.5273 (5)	0.0211 (5)	0.14145 (14)	0.071 (2)	
H43A	0.4436	0.0265	0.1316	0.086*	
H43B	0.5223	−0.0329	0.1531	0.086*	
C44	0.6406 (4)	0.0110 (4)	0.12176 (11)	0.0540 (15)	
H44A	0.7155	−0.0157	0.1319	0.065*	
C45	0.6241 (6)	0.2667 (4)	0.15108 (12)	0.073 (2)	
H45A	0.5590	0.2883	0.1639	0.110*	
H45B	0.6365	0.3123	0.1371	0.110*	
H45C	0.7074	0.2561	0.1602	0.110*	
C46	0.6139 (6)	−0.0510 (4)	0.09844 (15)	0.076 (2)	
H46A	0.5921	−0.1116	0.1050	0.115*	
H46B	0.6919	−0.0544	0.0871	0.115*	
H46C	0.5405	−0.0272	0.0880	0.115*	
C47	1.0193 (5)	0.1383 (5)	0.04934 (12)	0.0551 (17)	
H47A	0.9769	0.1515	0.0318	0.066*	
C48	1.1320 (6)	0.2034 (5)	0.05267 (15)	0.081 (2)	
H48A	1.1901	0.1832	0.0673	0.097*	
H48B	1.1838	0.2069	0.0360	0.097*	
C49	1.0735 (7)	0.2941 (5)	0.05912 (15)	0.081 (3)	
H49A	1.1432	0.3369	0.0645	0.098*	
H49B	1.0288	0.3190	0.0432	0.098*	
C50	0.9773 (5)	0.2826 (4)	0.08145 (12)	0.0561 (17)	
H50A	1.0296	0.2776	0.0982	0.067*	
C51	1.0593 (6)	0.0383 (5)	0.04937 (14)	0.075 (2)	
H51A	1.1193	0.0268	0.0345	0.113*	
H51B	0.9812	0.0004	0.0474	0.113*	
H51C	1.1028	0.0238	0.0662	0.113*	
C52	0.8835 (7)	0.3609 (4)	0.08534 (14)	0.085 (2)	
H52A	0.9331	0.4165	0.0886	0.127*	
H52B	0.8266	0.3484	0.1006	0.127*	
H52C	0.8299	0.3683	0.0693	0.127*	
C61	0.2458 (6)	0.5608 (4)	0.12921 (12)	0.0613 (18)	
H61A	0.1488	0.5543	0.1310	0.074*	
C62	0.2952 (6)	0.6401 (4)	0.14108 (11)	0.0612 (17)	
H62A	0.2276	0.6794	0.1497	0.073*	
C63	0.4270 (6)	0.6461 (6)	0.15322 (12)	0.086 (2)	
H63A	0.4506	0.5862	0.1606	0.103*	
H63B	0.4241	0.6897	0.1682	0.103*	
C64	0.5324 (6)	0.6755 (6)	0.13371 (12)	0.087 (2)	
H64A	0.5367	0.7424	0.1338	0.105*	
H64B	0.6176	0.6527	0.1403	0.105*	
C65	0.5158 (5)	0.6449 (5)	0.10556 (12)	0.0596 (19)	
H65A	0.5723	0.6777	0.0925	0.072*	
C66	0.4782 (6)	0.5581 (4)	0.09725 (14)	0.0636 (18)	
H66A	0.5112	0.5410	0.0792	0.076*	
C67	0.4544 (6)	0.4795 (5)	0.11517 (14)	0.086 (2)	
H67A	0.5153	0.4835	0.1305	0.104*	
H67B	0.4750	0.4232	0.1053	0.104*	
C68	0.3148 (7)	0.4730 (4)	0.12582 (14)	0.091 (2)	
H68A	0.2631	0.4348	0.1135	0.109*	
H68B	0.3171	0.4418	0.1433	0.109*	
C69	0.0849 (5)	0.7563 (4)	0.05786 (11)	0.0496 (16)	
H69A	0.0449	0.7027	0.0493	0.060*	
H69B	0.0240	0.8080	0.0560	0.060*	
C70	0.2147 (5)	0.7783 (4)	0.04481 (12)	0.0558 (16)	
H70A	0.2495	0.8362	0.0517	0.067*	
H70B	0.2040	0.7836	0.0253	0.067*	
C71	−0.0378 (5)	0.6989 (4)	0.10800 (11)	0.0463 (15)	
H71A	−0.0154	0.6674	0.1250	0.056*	
C72	−0.1042 (5)	0.7865 (4)	0.11559 (13)	0.0587 (18)	
H72A	−0.1437	0.8153	0.0997	0.070*	
H72B	−0.1740	0.7752	0.1289	0.070*	
C73	−0.0009 (5)	0.8454 (4)	0.12709 (12)	0.0633 (16)	
H73A	0.0264	0.8218	0.1447	0.076*	
H73B	−0.0353	0.9075	0.1296	0.076*	
C74	0.1158 (5)	0.8475 (4)	0.10829 (12)	0.0570 (16)	
H74A	0.0934	0.8912	0.0938	0.068*	
C75	−0.1210 (5)	0.6336 (5)	0.09196 (15)	0.088 (3)	
H75A	−0.2000	0.6183	0.1021	0.131*	
H75B	−0.0709	0.5783	0.0884	0.131*	
H75C	−0.1459	0.6619	0.0750	0.131*	
C76	0.2408 (6)	0.8800 (4)	0.12044 (16)	0.092 (3)	
H76A	0.2282	0.9410	0.1276	0.138*	
H76B	0.3094	0.8813	0.1068	0.138*	
H76C	0.2668	0.8389	0.1348	0.138*	
C77	0.4794 (5)	0.7060 (4)	0.03511 (12)	0.0466 (15)	
H77A	0.5483	0.6686	0.0440	0.056*	
C78	0.4552 (5)	0.6657 (4)	0.00776 (11)	0.0618 (16)	
H78A	0.4007	0.7070	−0.0031	0.074*	
H78B	0.5391	0.6557	−0.0016	0.074*	
C79	0.3853 (6)	0.5768 (4)	0.01195 (13)	0.0663 (19)	
H79A	0.4455	0.5320	0.0200	0.080*	
H79B	0.3540	0.5527	−0.0053	0.080*	
C80	0.2696 (4)	0.5941 (4)	0.03052 (11)	0.0470 (15)	
H80A	0.1958	0.6179	0.0195	0.056*	
C81	0.5255 (6)	0.8022 (4)	0.03515 (14)	0.074 (2)	
H81A	0.6094	0.8062	0.0258	0.111*	
H81B	0.5365	0.8228	0.0536	0.111*	
H81C	0.4609	0.8404	0.0261	0.111*	
C82	0.2231 (5)	0.5091 (4)	0.04417 (11)	0.0670 (17)	
H82A	0.1949	0.4651	0.0307	0.101*	
H82B	0.1495	0.5236	0.0559	0.101*	
H82C	0.2947	0.4833	0.0547	0.101*	
B1	0.7819 (5)	0.7243 (5)	0.35410 (13)	0.0457 (17)	
F1	0.7859 (3)	0.6327 (2)	0.35020 (7)	0.0892 (12)	
F2	0.7065 (3)	0.7637 (2)	0.33541 (6)	0.0820 (11)	
F3	0.9076 (2)	0.7578 (2)	0.35287 (7)	0.0726 (11)	
F4	0.7329 (3)	0.7410 (2)	0.37907 (6)	0.0874 (12)	
B3	0.6346 (7)	0.8352 (6)	0.19316 (15)	0.061 (2)	
F9	0.7009 (3)	0.8256 (3)	0.16969 (6)	0.0978 (12)	
F10	0.5025 (3)	0.8416 (3)	0.18800 (7)	0.0834 (11)	
F11	0.6560 (4)	0.7609 (3)	0.20857 (9)	0.1390 (19)	
F12	0.6746 (4)	0.9104 (3)	0.20584 (8)	0.1192 (17)	
B2	0.7930 (8)	0.5482 (7)	0.02251 (15)	0.100 (4)	
F5	0.9082 (5)	0.5195 (5)	0.03039 (12)	0.090 (3)*	0.618 (7)
F6	0.7925 (6)	0.6469 (3)	0.02415 (12)	0.104 (3)*	0.618 (7)
F7	0.6990 (6)	0.5245 (4)	0.04215 (11)	0.073 (3)*	0.618 (7)
F8	0.7524 (7)	0.5256 (5)	−0.00082 (11)	0.125 (4)*	0.618 (7)
F5'	0.8285 (11)	0.4399 (5)	0.0204 (2)	0.126 (5)*	0.382 (7)
F6'	0.9221 (9)	0.5735 (7)	0.0278 (2)	0.094 (4)*	0.382 (7)
F7'	0.7174 (11)	0.5484 (9)	0.0412 (2)	0.107 (6)*	0.382 (7)
F8'	0.7640 (8)	0.5615 (7)	−0.00230 (17)	0.072 (4)*	0.382 (7)

### Atomic displacement parameters (Å^2^)

**Table:**

	*U*^11^	*U*^22^	*U*^33^	*U*^12^	*U*^13^	*U*^23^
Rh1	0.02789 (17)	0.0373 (2)	0.02778 (19)	0.0001 (2)	0.00228 (18)	0.00123 (18)
Rh2	0.0306 (2)	0.0494 (3)	0.0293 (2)	0.0028 (2)	−0.0029 (2)	−0.0004 (2)
Rh3	0.0333 (2)	0.0494 (3)	0.0278 (2)	0.0021 (2)	0.00166 (19)	0.00467 (19)
P1	0.0351 (6)	0.0387 (8)	0.0264 (8)	−0.0014 (6)	−0.0027 (5)	−0.0014 (6)
P2	0.0310 (6)	0.0492 (10)	0.0325 (8)	−0.0029 (6)	0.0030 (6)	−0.0011 (7)
P21	0.0333 (7)	0.0506 (9)	0.0355 (8)	−0.0013 (8)	−0.0026 (8)	0.0032 (6)
P22	0.0322 (7)	0.0491 (10)	0.0349 (9)	−0.0039 (7)	−0.0032 (6)	0.0045 (8)
P31	0.0339 (7)	0.0499 (10)	0.0357 (9)	0.0022 (7)	0.0049 (6)	0.0023 (7)
P32	0.0340 (7)	0.0477 (8)	0.0299 (7)	0.0041 (8)	0.0038 (7)	0.0052 (6)
C1	0.028 (3)	0.061 (4)	0.044 (4)	0.006 (3)	−0.005 (2)	−0.002 (3)
C2	0.034 (3)	0.065 (5)	0.040 (4)	−0.011 (3)	0.009 (2)	−0.002 (3)
C5	0.037 (4)	0.107 (6)	0.043 (4)	0.002 (4)	−0.001 (3)	0.027 (4)
C6	0.030 (3)	0.127 (6)	0.033 (4)	0.018 (4)	−0.004 (2)	−0.014 (4)
C9	0.051 (3)	0.046 (3)	0.029 (3)	−0.002 (3)	0.002 (2)	−0.004 (2)
C10	0.036 (2)	0.046 (4)	0.035 (3)	0.001 (3)	0.011 (2)	0.000 (3)
C11	0.041 (3)	0.043 (4)	0.042 (3)	0.004 (3)	−0.005 (2)	−0.007 (3)
C12	0.050 (3)	0.062 (4)	0.057 (4)	0.002 (3)	−0.018 (3)	−0.016 (3)
C13	0.058 (4)	0.046 (4)	0.068 (5)	−0.005 (3)	−0.013 (3)	−0.006 (3)
C14	0.052 (3)	0.040 (3)	0.036 (3)	0.001 (3)	−0.001 (2)	−0.003 (2)
C15	0.081 (5)	0.059 (5)	0.053 (5)	0.019 (4)	−0.021 (3)	0.009 (3)
C16	0.080 (4)	0.040 (4)	0.066 (5)	0.001 (3)	0.007 (4)	−0.007 (3)
C17	0.033 (3)	0.087 (5)	0.046 (4)	−0.001 (4)	0.001 (2)	−0.007 (4)
C18	0.042 (3)	0.110 (6)	0.059 (5)	−0.015 (4)	0.000 (3)	−0.010 (4)
C19	0.052 (3)	0.088 (5)	0.064 (4)	−0.027 (4)	0.008 (3)	−0.026 (4)
C20	0.051 (3)	0.058 (4)	0.047 (4)	−0.012 (3)	0.017 (3)	−0.005 (3)
C21	0.057 (4)	0.102 (6)	0.066 (5)	0.023 (4)	−0.009 (4)	0.012 (4)
C22	0.077 (4)	0.044 (3)	0.087 (5)	−0.006 (3)	0.015 (4)	−0.008 (3)
C31	0.041 (3)	0.063 (4)	0.038 (4)	0.023 (3)	−0.001 (3)	−0.002 (3)
C32	0.021 (3)	0.080 (5)	0.048 (4)	0.004 (3)	−0.001 (2)	−0.010 (4)
C33	0.051 (4)	0.137 (7)	0.060 (5)	−0.014 (4)	−0.007 (3)	−0.036 (5)
C34	0.065 (5)	0.149 (8)	0.068 (6)	−0.011 (5)	0.014 (4)	−0.052 (5)
C35	0.037 (3)	0.106 (5)	0.033 (3)	0.000 (4)	0.000 (3)	−0.015 (3)
C36	0.051 (3)	0.096 (5)	0.031 (3)	0.010 (4)	−0.004 (3)	0.019 (3)
C37	0.076 (5)	0.134 (7)	0.061 (5)	0.046 (5)	0.006 (4)	0.036 (5)
C38	0.093 (5)	0.095 (6)	0.057 (5)	0.047 (4)	−0.002 (4)	0.022 (4)
C39	0.044 (3)	0.064 (4)	0.029 (3)	0.000 (3)	−0.003 (2)	0.014 (3)
C40	0.027 (3)	0.053 (4)	0.045 (4)	−0.007 (3)	−0.002 (2)	0.009 (3)
C41	0.039 (3)	0.067 (4)	0.037 (3)	0.002 (3)	0.000 (2)	0.005 (3)
C42	0.054 (4)	0.101 (6)	0.049 (5)	0.000 (4)	0.009 (3)	0.021 (4)
C43	0.056 (4)	0.074 (5)	0.085 (6)	−0.008 (4)	0.013 (3)	0.030 (4)
C44	0.037 (3)	0.060 (4)	0.065 (4)	0.006 (3)	−0.002 (2)	0.013 (3)
C45	0.073 (4)	0.104 (6)	0.044 (4)	−0.003 (4)	0.001 (3)	−0.015 (4)
C46	0.065 (4)	0.041 (4)	0.124 (7)	−0.011 (3)	−0.012 (4)	0.015 (4)
C47	0.038 (3)	0.088 (5)	0.040 (4)	0.001 (3)	0.000 (2)	0.006 (3)
C48	0.041 (4)	0.124 (7)	0.078 (6)	−0.009 (4)	0.005 (3)	0.022 (5)
C49	0.067 (4)	0.103 (7)	0.075 (6)	−0.033 (4)	−0.009 (4)	0.042 (5)
C50	0.055 (4)	0.057 (4)	0.057 (4)	−0.015 (3)	−0.008 (3)	0.022 (3)
C51	0.060 (4)	0.111 (7)	0.055 (5)	0.017 (4)	0.005 (3)	−0.021 (4)
C52	0.107 (6)	0.047 (4)	0.100 (7)	−0.003 (4)	−0.015 (4)	−0.004 (4)
C61	0.067 (4)	0.070 (5)	0.047 (4)	−0.003 (3)	0.015 (3)	0.025 (3)
C62	0.064 (4)	0.087 (5)	0.032 (3)	−0.008 (4)	0.006 (3)	0.009 (3)
C63	0.086 (5)	0.138 (7)	0.034 (4)	−0.009 (5)	−0.004 (3)	0.001 (4)
C64	0.058 (4)	0.149 (8)	0.055 (5)	−0.010 (5)	−0.018 (3)	0.012 (5)
C65	0.034 (3)	0.097 (6)	0.048 (4)	0.005 (4)	−0.011 (3)	0.007 (4)
C66	0.064 (4)	0.074 (5)	0.053 (5)	0.028 (4)	0.008 (3)	0.011 (4)
C67	0.113 (6)	0.077 (6)	0.069 (5)	0.049 (5)	0.011 (4)	0.025 (4)
C68	0.103 (5)	0.063 (5)	0.106 (6)	0.015 (5)	0.004 (5)	0.024 (4)
C69	0.043 (3)	0.070 (5)	0.037 (4)	0.011 (3)	0.002 (3)	0.009 (3)
C70	0.064 (4)	0.063 (4)	0.041 (4)	0.014 (3)	0.009 (3)	0.020 (3)
C71	0.037 (3)	0.053 (4)	0.049 (4)	0.007 (3)	0.008 (2)	−0.003 (3)
C72	0.041 (3)	0.081 (5)	0.055 (4)	0.022 (3)	0.018 (3)	0.005 (3)
C73	0.073 (4)	0.063 (4)	0.054 (4)	0.014 (4)	0.016 (3)	−0.002 (4)
C74	0.056 (3)	0.044 (4)	0.071 (5)	−0.002 (3)	0.009 (3)	0.005 (3)
C75	0.047 (4)	0.113 (7)	0.103 (7)	−0.010 (4)	0.019 (4)	−0.039 (5)
C76	0.077 (5)	0.067 (5)	0.132 (8)	−0.012 (4)	−0.004 (4)	−0.041 (4)
C77	0.044 (3)	0.047 (4)	0.048 (4)	−0.001 (3)	0.003 (3)	−0.003 (3)
C78	0.064 (4)	0.078 (5)	0.043 (4)	0.000 (4)	0.020 (3)	0.004 (4)
C79	0.063 (4)	0.090 (5)	0.046 (4)	−0.011 (4)	0.014 (3)	−0.025 (4)
C80	0.036 (3)	0.069 (4)	0.037 (4)	−0.004 (3)	−0.002 (2)	−0.004 (3)
C81	0.073 (4)	0.075 (5)	0.074 (5)	−0.014 (4)	0.023 (4)	−0.002 (4)
C82	0.076 (4)	0.068 (4)	0.058 (4)	−0.011 (4)	−0.003 (3)	−0.009 (4)
B1	0.035 (4)	0.062 (5)	0.040 (4)	0.000 (3)	−0.002 (3)	0.013 (3)
F1	0.090 (3)	0.073 (3)	0.104 (3)	−0.003 (2)	−0.010 (2)	−0.001 (2)
F2	0.066 (2)	0.110 (3)	0.070 (3)	0.011 (2)	−0.019 (2)	0.023 (2)
F3	0.0399 (19)	0.110 (3)	0.068 (3)	−0.004 (2)	0.0011 (17)	0.032 (2)
F4	0.068 (2)	0.142 (4)	0.052 (3)	−0.012 (2)	0.0107 (17)	−0.010 (2)
B3	0.059 (5)	0.076 (6)	0.049 (5)	0.010 (5)	0.007 (3)	0.006 (5)
F9	0.095 (2)	0.145 (4)	0.053 (2)	0.026 (3)	0.021 (2)	−0.015 (2)
F10	0.070 (2)	0.092 (3)	0.089 (3)	0.008 (2)	0.0092 (19)	−0.009 (2)
F11	0.136 (4)	0.138 (4)	0.143 (5)	0.039 (4)	0.004 (3)	0.062 (3)
F12	0.073 (3)	0.151 (4)	0.133 (4)	−0.025 (3)	0.021 (3)	−0.068 (3)
B2	0.073 (6)	0.194 (12)	0.034 (5)	0.046 (6)	0.002 (4)	−0.024 (5)

### Geometric parameters (Å, °)

**Table:**

Rh1—C5	2.163 (6)	C35—C36	1.399 (4)
Rh1—C1	2.209 (5)	C35—H35A	1.0000
Rh1—C2	2.232 (5)	C36—C37	1.486 (8)
Rh1—C6	2.236 (5)	C36—H36A	1.0000
Rh1—P2	2.2737 (13)	C37—C38	1.513 (4)
Rh1—P1	2.2786 (14)	C37—H37A	0.9900
Rh2—C35	2.215 (5)	C37—H37B	0.9900
Rh2—C31	2.218 (5)	C38—H38A	0.9900
Rh2—C36	2.229 (5)	C38—H38B	0.9900
Rh2—C32	2.259 (5)	C39—C40	1.518 (7)
Rh2—P22	2.2602 (14)	C39—H39A	0.9900
Rh2—P21	2.2702 (15)	C39—H39B	0.9900
Rh3—C65	2.200 (5)	C40—H40A	0.9900
Rh3—C61	2.205 (5)	C40—H40B	0.9900
Rh3—C62	2.239 (5)	C41—C42	1.503 (8)
Rh3—C66	2.249 (6)	C41—C45	1.506 (8)
Rh3—P32	2.2642 (14)	C41—H41A	1.0000
Rh3—P31	2.2797 (14)	C42—C43	1.491 (9)
P1—C11	1.830 (5)	C42—H42A	0.9900
P1—C9	1.837 (4)	C42—H42B	0.9900
P1—C14	1.853 (5)	C43—C44	1.530 (7)
P2—C10	1.821 (5)	C43—H43A	0.9900
P2—C17	1.843 (5)	C43—H43B	0.9900
P2—C20	1.852 (6)	C44—C46	1.510 (8)
P21—C39	1.825 (5)	C44—H44A	1.0000
P21—C41	1.837 (5)	C45—H45A	0.9800
P21—C44	1.847 (6)	C45—H45B	0.9800
P22—C40	1.819 (5)	C45—H45C	0.9800
P22—C47	1.832 (6)	C46—H46A	0.9800
P22—C50	1.834 (6)	C46—H46B	0.9800
P31—C69	1.820 (6)	C46—H46C	0.9800
P31—C71	1.826 (5)	C47—C48	1.510 (8)
P31—C74	1.850 (6)	C47—C51	1.533 (8)
P32—C70	1.814 (5)	C47—H47A	1.0000
P32—C77	1.832 (5)	C48—C49	1.502 (9)
P32—C80	1.850 (5)	C48—H48A	0.9900
C1—C2	1.398 (4)	C48—H48B	0.9900
C1—C8'	1.479 (12)	C49—C50	1.500 (8)
C1—C8	1.530 (13)	C49—H49A	0.9900
C1—H1A	1.0000	C49—H49B	0.9900
C1—H1B	1.0000	C50—C52	1.515 (8)
C2—C3	1.416 (12)	C50—H50A	1.0000
C2—C3'	1.614 (14)	C51—H51A	0.9800
C2—H2A	1.0000	C51—H51B	0.9800
C2—H2B	1.0000	C51—H51C	0.9800
C3—C4	1.535 (5)	C52—H52A	0.9800
C3—H3A	0.9900	C52—H52B	0.9800
C3—H3B	0.9900	C52—H52C	0.9800
C4—C5	1.641 (12)	C61—C62	1.409 (4)
C4—H4A	0.9900	C61—C68	1.486 (7)
C4—H4B	0.9900	C61—H61A	1.0000
C3'—C4'	1.529 (5)	C62—C63	1.481 (8)
C3'—H3C	0.9900	C62—H62A	1.0000
C3'—H3D	0.9900	C63—C64	1.520 (4)
C4'—C5	1.439 (12)	C63—H63A	0.9900
C4'—H4C	0.9900	C63—H63B	0.9900
C4'—H4D	0.9900	C64—C65	1.492 (8)
C5—C6	1.410 (4)	C64—H64A	0.9900
C5—H5A	1.0000	C64—H64B	0.9900
C5—H5B	1.0000	C65—C66	1.402 (5)
C6—C7	1.514 (11)	C65—H65A	1.0000
C6—C7'	1.564 (11)	C66—C67	1.488 (8)
C6—H6A	1.0000	C66—H66A	1.0000
C6—H6B	1.0000	C67—C68	1.527 (5)
C7—C8	1.542 (5)	C67—H67A	0.9900
C7—H7A	0.9900	C67—H67B	0.9900
C7—H7B	0.9900	C68—H68A	0.9900
C8—H8A	0.9900	C68—H68B	0.9900
C8—H8B	0.9900	C69—C70	1.515 (7)
C7'—C8'	1.536 (5)	C69—H69A	0.9900
C7'—H7C	0.9900	C69—H69B	0.9900
C7'—H7D	0.9900	C70—H70A	0.9900
C8'—H8C	0.9900	C70—H70B	0.9900
C8'—H8D	0.9900	C71—C72	1.509 (7)
C9—C10	1.501 (6)	C71—C75	1.517 (7)
C9—H9A	0.9900	C71—H71A	1.0000
C9—H9B	0.9900	C72—C73	1.486 (8)
C10—H10A	0.9900	C72—H72A	0.9900
C10—H10B	0.9900	C72—H72B	0.9900
C11—C12	1.506 (7)	C73—C74	1.521 (7)
C11—C15	1.509 (8)	C73—H73A	0.9900
C11—H11A	1.0000	C73—H73B	0.9900
C12—C13	1.523 (8)	C74—C76	1.496 (7)
C12—H12A	0.9900	C74—H74A	1.0000
C12—H12B	0.9900	C75—H75A	0.9800
C13—C14	1.529 (7)	C75—H75B	0.9800
C13—H13A	0.9900	C75—H75C	0.9800
C13—H13B	0.9900	C76—H76A	0.9800
C14—C16	1.509 (7)	C76—H76B	0.9800
C14—H14A	1.0000	C76—H76C	0.9800
C15—H15A	0.9800	C77—C81	1.497 (7)
C15—H15B	0.9800	C77—C78	1.516 (8)
C15—H15C	0.9800	C77—H77A	1.0000
C16—H16A	0.9800	C78—C79	1.509 (8)
C16—H16B	0.9800	C78—H78A	0.9900
C16—H16C	0.9800	C78—H78B	0.9900
C17—C21	1.497 (8)	C79—C80	1.527 (7)
C17—C18	1.527 (8)	C79—H79A	0.9900
C17—H17A	1.0000	C79—H79B	0.9900
C18—C19	1.505 (8)	C80—C82	1.507 (7)
C18—H18A	0.9900	C80—H80A	1.0000
C18—H18B	0.9900	C81—H81A	0.9800
C19—C20	1.528 (7)	C81—H81B	0.9800
C19—H19A	0.9900	C81—H81C	0.9800
C19—H19B	0.9900	C82—H82A	0.9800
C20—C22	1.503 (7)	C82—H82B	0.9800
C20—H20A	1.0000	C82—H82C	0.9800
C21—H21A	0.9800	B1—F2	1.346 (6)
C21—H21B	0.9800	B1—F1	1.368 (7)
C21—H21C	0.9800	B1—F4	1.371 (7)
C22—H22A	0.9800	B1—F3	1.379 (6)
C22—H22B	0.9800	B3—F12	1.344 (8)
C22—H22C	0.9800	B3—F11	1.360 (8)
C31—C32	1.403 (4)	B3—F9	1.366 (7)
C31—C38	1.476 (8)	B3—F10	1.378 (7)
C31—H31A	1.0000	B2—F7'	1.213 (10)
C32—C33	1.489 (7)	B2—F8	1.285 (9)
C32—H32A	1.0000	B2—F8'	1.294 (11)
C33—C34	1.510 (4)	B2—F5	1.313 (9)
C33—H33A	0.9900	B2—F6'	1.398 (11)
C33—H33B	0.9900	B2—F7	1.419 (9)
C34—C35	1.464 (8)	B2—F6	1.460 (10)
C34—H34A	0.9900	B2—F5'	1.644 (12)
C34—H34B	0.9900		
			
C5—Rh1—C1	92.4 (2)	C32—C33—H33B	108.4
C5—Rh1—C2	81.2 (2)	C34—C33—H33B	108.4
C1—Rh1—C2	36.70 (12)	H33A—C33—H33B	107.4
C5—Rh1—C6	37.33 (13)	C35—C34—C33	116.7 (5)
C1—Rh1—C6	79.8 (2)	C35—C34—H34A	108.1
C2—Rh1—C6	91.7 (2)	C33—C34—H34A	108.1
C5—Rh1—P2	98.51 (15)	C35—C34—H34B	108.1
C1—Rh1—P2	153.42 (15)	C33—C34—H34B	108.1
C2—Rh1—P2	169.37 (14)	H34A—C34—H34B	107.3
C6—Rh1—P2	94.43 (15)	C36—C35—C34	127.6 (7)
C5—Rh1—P1	150.27 (19)	C36—C35—Rh2	72.2 (3)
C1—Rh1—P1	99.46 (15)	C34—C35—Rh2	107.0 (4)
C2—Rh1—P1	92.23 (15)	C36—C35—H35A	113.7
C6—Rh1—P1	172.15 (19)	C34—C35—H35A	113.7
P2—Rh1—P1	82.78 (5)	Rh2—C35—H35A	113.7
C35—Rh2—C31	94.5 (2)	C35—C36—C37	124.1 (7)
C35—Rh2—C36	36.70 (12)	C35—C36—Rh2	71.1 (3)
C31—Rh2—C36	80.1 (2)	C37—C36—Rh2	111.5 (4)
C35—Rh2—C32	80.8 (2)	C35—C36—H36A	114.2
C31—Rh2—C32	36.52 (12)	C37—C36—H36A	114.2
C36—Rh2—C32	88.9 (2)	Rh2—C36—H36A	114.2
C35—Rh2—P22	96.78 (17)	C36—C37—C38	113.8 (5)
C31—Rh2—P22	153.41 (15)	C36—C37—H37A	108.8
C36—Rh2—P22	94.91 (18)	C38—C37—H37A	108.8
C32—Rh2—P22	169.96 (15)	C36—C37—H37B	108.8
C35—Rh2—P21	150.21 (16)	C38—C37—H37B	108.8
C31—Rh2—P21	98.74 (16)	H37A—C37—H37B	107.7
C36—Rh2—P21	172.88 (16)	C31—C38—C37	116.2 (5)
C32—Rh2—P21	94.30 (16)	C31—C38—H38A	108.2
P22—Rh2—P21	82.97 (6)	C37—C38—H38A	108.2
C65—Rh3—C61	95.1 (2)	C31—C38—H38B	108.2
C65—Rh3—C62	80.7 (2)	C37—C38—H38B	108.2
C61—Rh3—C62	36.95 (13)	H38A—C38—H38B	107.4
C65—Rh3—C66	36.71 (13)	C40—C39—P21	107.4 (4)
C61—Rh3—C66	80.1 (2)	C40—C39—H39A	110.2
C62—Rh3—C66	88.5 (2)	P21—C39—H39A	110.2
C65—Rh3—P32	96.94 (17)	C40—C39—H39B	110.2
C61—Rh3—P32	151.68 (16)	P21—C39—H39B	110.2
C62—Rh3—P32	171.30 (16)	H39A—C39—H39B	108.5
C66—Rh3—P32	94.59 (18)	C39—C40—P22	106.2 (4)
C65—Rh3—P31	150.34 (18)	C39—C40—H40A	110.5
C61—Rh3—P31	98.63 (16)	P22—C40—H40A	110.5
C62—Rh3—P31	94.82 (17)	C39—C40—H40B	110.5
C66—Rh3—P31	172.70 (18)	P22—C40—H40B	110.5
P32—Rh3—P31	83.09 (5)	H40A—C40—H40B	108.7
C11—P1—C9	107.7 (2)	C42—C41—C45	115.5 (5)
C11—P1—C14	94.4 (2)	C42—C41—P21	104.2 (4)
C9—P1—C14	103.1 (2)	C45—C41—P21	115.9 (4)
C11—P1—Rh1	122.41 (18)	C42—C41—H41A	106.8
C9—P1—Rh1	109.67 (15)	C45—C41—H41A	106.8
C14—P1—Rh1	117.27 (17)	P21—C41—H41A	106.8
C10—P2—C17	108.2 (2)	C43—C42—C41	106.6 (6)
C10—P2—C20	104.7 (2)	C43—C42—H42A	110.4
C17—P2—C20	95.0 (3)	C41—C42—H42A	110.4
C10—P2—Rh1	109.00 (15)	C43—C42—H42B	110.4
C17—P2—Rh1	124.76 (19)	C41—C42—H42B	110.4
C20—P2—Rh1	113.03 (15)	H42A—C42—H42B	108.6
C39—P21—C41	106.2 (3)	C42—C43—C44	108.7 (5)
C39—P21—C44	103.2 (2)	C42—C43—H43A	110.0
C41—P21—C44	94.8 (3)	C44—C43—H43A	110.0
C39—P21—Rh2	109.54 (17)	C42—C43—H43B	110.0
C41—P21—Rh2	122.78 (19)	C44—C43—H43B	110.0
C44—P21—Rh2	118.00 (19)	H43A—C43—H43B	108.3
C40—P22—C47	107.9 (3)	C46—C44—C43	115.0 (5)
C40—P22—C50	104.1 (3)	C46—C44—P21	116.5 (4)
C47—P22—C50	95.1 (3)	C43—C44—P21	103.9 (4)
C40—P22—Rh2	109.13 (16)	C46—C44—H44A	107.0
C47—P22—Rh2	123.43 (19)	C43—C44—H44A	107.0
C50—P22—Rh2	115.00 (19)	P21—C44—H44A	107.0
C69—P31—C71	107.3 (3)	C41—C45—H45A	109.5
C69—P31—C74	103.5 (3)	C41—C45—H45B	109.5
C71—P31—C74	94.4 (2)	H45A—C45—H45B	109.5
C69—P31—Rh3	109.15 (18)	C41—C45—H45C	109.5
C71—P31—Rh3	123.37 (19)	H45A—C45—H45C	109.5
C74—P31—Rh3	116.79 (19)	H45B—C45—H45C	109.5
C70—P32—C77	108.0 (3)	C44—C46—H46A	109.5
C70—P32—C80	103.1 (3)	C44—C46—H46B	109.5
C77—P32—C80	95.1 (2)	H46A—C46—H46B	109.5
C70—P32—Rh3	108.96 (18)	C44—C46—H46C	109.5
C77—P32—Rh3	125.7 (2)	H46A—C46—H46C	109.5
C80—P32—Rh3	113.30 (19)	H46B—C46—H46C	109.5
C2—C1—C8'	118.7 (7)	C48—C47—C51	114.2 (5)
C2—C1—C8	134.5 (7)	C48—C47—P22	104.1 (4)
C2—C1—Rh1	72.6 (3)	C51—C47—P22	115.1 (4)
C8'—C1—Rh1	113.4 (4)	C48—C47—H47A	107.7
C8—C1—Rh1	105.3 (5)	C51—C47—H47A	107.7
C2—C1—H1A	111.4	P22—C47—H47A	107.7
C8'—C1—H1A	120.0	C49—C48—C47	106.7 (5)
C8—C1—H1A	111.4	C49—C48—H48A	110.4
Rh1—C1—H1A	111.4	C47—C48—H48A	110.4
C2—C1—H1B	114.7	C49—C48—H48B	110.4
C8'—C1—H1B	115.1	C47—C48—H48B	110.4
C8—C1—H1B	107.1	H48A—C48—H48B	108.6
Rh1—C1—H1B	115.5	C50—C49—C48	108.7 (5)
C1—C2—C3	118.8 (8)	C50—C49—H49A	110.0
C1—C2—C3'	128.7 (7)	C48—C49—H49A	110.0
C1—C2—Rh1	70.8 (3)	C50—C49—H49B	110.0
C3—C2—Rh1	114.1 (5)	C48—C49—H49B	109.9
C3'—C2—Rh1	101.6 (6)	H49A—C49—H49B	108.3
C1—C2—H2A	115.3	C49—C50—C52	115.1 (5)
C3—C2—H2A	115.3	C49—C50—P22	104.5 (5)
C3'—C2—H2A	113.6	C52—C50—P22	116.6 (4)
Rh1—C2—H2A	115.3	C49—C50—H50A	106.6
C1—C2—H2B	114.3	C52—C50—H50A	106.6
C3—C2—H2B	116.9	P22—C50—H50A	106.6
Rh1—C2—H2B	113.6	C47—C51—H51A	109.5
C2—C3—C4	111.4 (8)	C47—C51—H51B	109.5
C2—C3—H3A	109.4	H51A—C51—H51B	109.5
C4—C3—H3A	109.4	C47—C51—H51C	109.5
C2—C3—H3B	109.4	H51A—C51—H51C	109.5
C4—C3—H3B	109.4	H51B—C51—H51C	109.5
H3A—C3—H3B	108.0	C50—C52—H52A	109.5
C3—C4—C5	111.6 (8)	C50—C52—H52B	109.5
C3—C4—H4A	109.3	H52A—C52—H52B	109.5
C5—C4—H4A	109.3	C50—C52—H52C	109.5
C3—C4—H4B	109.3	H52A—C52—H52C	109.5
C5—C4—H4B	109.3	H52B—C52—H52C	109.5
H4A—C4—H4B	108.0	C62—C61—C68	127.2 (6)
C4'—C3'—C2	114.9 (10)	C62—C61—Rh3	72.8 (3)
C4'—C3'—H3C	108.6	C68—C61—Rh3	108.5 (4)
C2—C3'—H3C	108.6	C62—C61—H61A	113.4
C4'—C3'—H3D	108.6	C68—C61—H61A	113.4
C2—C3'—H3D	108.6	Rh3—C61—H61A	113.4
H3C—C3'—H3D	107.5	C61—C62—C63	123.4 (6)
C5—C4'—C3'	107.2 (10)	C61—C62—Rh3	70.2 (3)
C5—C4'—H4C	110.3	C63—C62—Rh3	111.3 (4)
C3'—C4'—H4C	110.3	C61—C62—H62A	114.6
C5—C4'—H4D	110.3	C63—C62—H62A	114.6
C3'—C4'—H4D	110.3	Rh3—C62—H62A	114.6
H4C—C4'—H4D	108.5	C62—C63—C64	113.4 (5)
C6—C5—C4'	108.9 (8)	C62—C63—H63A	108.9
C6—C5—C4	133.4 (6)	C64—C63—H63A	108.9
C6—C5—Rh1	74.2 (3)	C62—C63—H63B	108.9
C4—C5—Rh1	103.0 (5)	C64—C63—H63B	108.9
C6—C5—H5A	111.9	H63A—C63—H63B	107.7
C4'—C5—H5A	122.2	C65—C64—C63	116.2 (6)
C4—C5—H5A	111.9	C65—C64—H64A	108.2
Rh1—C5—H5A	111.9	C63—C64—H64A	108.2
C6—C5—H5B	116.4	C65—C64—H64B	108.2
C4'—C5—H5B	115.8	C63—C64—H64B	108.2
C4—C5—H5B	106.3	H64A—C64—H64B	107.4
Rh1—C5—H5B	117.1	C66—C65—C64	126.2 (6)
C5—C6—C7	110.8 (7)	C66—C65—Rh3	73.5 (3)
C5—C6—C7'	137.7 (7)	C64—C65—Rh3	106.9 (4)
C5—C6—Rh1	68.5 (3)	C66—C65—H65A	114.0
C7—C6—Rh1	111.2 (5)	C64—C65—H65A	114.0
C7'—C6—Rh1	105.4 (5)	Rh3—C65—H65A	114.0
C5—C6—H6A	118.7	C65—C66—C67	125.3 (7)
C7—C6—H6A	118.7	C65—C66—Rh3	69.8 (3)
C7'—C6—H6A	101.2	C67—C66—Rh3	111.2 (4)
Rh1—C6—H6A	118.7	C65—C66—H66A	114.0
C5—C6—H6B	110.3	C67—C66—H66A	114.0
C7—C6—H6B	129.6	Rh3—C66—H66A	114.0
C7'—C6—H6B	110.9	C66—C67—C68	114.4 (5)
Rh1—C6—H6B	110.1	C66—C67—H67A	108.7
C6—C7—C8	107.4 (9)	C68—C67—H67A	108.7
C6—C7—H7A	110.2	C66—C67—H67B	108.7
C8—C7—H7A	110.2	C68—C67—H67B	108.7
C6—C7—H7B	110.2	H67A—C67—H67B	107.6
C8—C7—H7B	110.2	C61—C68—C67	115.4 (5)
H7A—C7—H7B	108.5	C61—C68—H68A	108.4
C1—C8—C7	109.7 (9)	C67—C68—H68A	108.4
C1—C8—H8A	109.7	C61—C68—H68B	108.4
C7—C8—H8A	109.7	C67—C68—H68B	108.4
C1—C8—H8B	109.7	H68A—C68—H68B	107.5
C7—C8—H8B	109.7	C70—C69—P31	107.4 (4)
H8A—C8—H8B	108.2	C70—C69—H69A	110.2
C8'—C7'—C6	110.8 (8)	P31—C69—H69A	110.2
C8'—C7'—H7C	109.5	C70—C69—H69B	110.2
C6—C7'—H7C	109.5	P31—C69—H69B	110.2
C8'—C7'—H7D	109.5	H69A—C69—H69B	108.5
C6—C7'—H7D	109.5	C69—C70—P32	106.9 (4)
H7C—C7'—H7D	108.1	C69—C70—H70A	110.3
C1—C8'—C7'	112.8 (8)	P32—C70—H70A	110.3
C1—C8'—H8C	109.0	C69—C70—H70B	110.3
C7'—C8'—H8C	109.0	P32—C70—H70B	110.3
C1—C8'—H8D	109.0	H70A—C70—H70B	108.6
C7'—C8'—H8D	109.0	C72—C71—C75	115.3 (5)
H8C—C8'—H8D	107.8	C72—C71—P31	104.7 (4)
C10—C9—P1	108.2 (3)	C75—C71—P31	116.4 (4)
C10—C9—H9A	110.1	C72—C71—H71A	106.6
P1—C9—H9A	110.1	C75—C71—H71A	106.6
C10—C9—H9B	110.1	P31—C71—H71A	106.6
P1—C9—H9B	110.1	C73—C72—C71	106.3 (4)
H9A—C9—H9B	108.4	C73—C72—H72A	110.5
C9—C10—P2	106.8 (3)	C71—C72—H72A	110.5
C9—C10—H10A	110.4	C73—C72—H72B	110.5
P2—C10—H10A	110.4	C71—C72—H72B	110.5
C9—C10—H10B	110.4	H72A—C72—H72B	108.7
P2—C10—H10B	110.4	C72—C73—C74	109.2 (5)
H10A—C10—H10B	108.6	C72—C73—H73A	109.8
C12—C11—C15	115.2 (5)	C74—C73—H73A	109.8
C12—C11—P1	105.9 (4)	C72—C73—H73B	109.8
C15—C11—P1	114.9 (4)	C74—C73—H73B	109.8
C12—C11—H11A	106.8	H73A—C73—H73B	108.3
C15—C11—H11A	106.8	C76—C74—C73	115.1 (5)
P1—C11—H11A	106.8	C76—C74—P31	117.2 (4)
C11—C12—C13	105.9 (5)	C73—C74—P31	104.2 (4)
C11—C12—H12A	110.6	C76—C74—H74A	106.5
C13—C12—H12A	110.6	C73—C74—H74A	106.5
C11—C12—H12B	110.6	P31—C74—H74A	106.5
C13—C12—H12B	110.6	C71—C75—H75A	109.5
H12A—C12—H12B	108.7	C71—C75—H75B	109.5
C12—C13—C14	108.8 (4)	H75A—C75—H75B	109.5
C12—C13—H13A	109.9	C71—C75—H75C	109.5
C14—C13—H13A	109.9	H75A—C75—H75C	109.5
C12—C13—H13B	109.9	H75B—C75—H75C	109.5
C14—C13—H13B	109.9	C74—C76—H76A	109.5
H13A—C13—H13B	108.3	C74—C76—H76B	109.5
C16—C14—C13	114.8 (4)	H76A—C76—H76B	109.5
C16—C14—P1	117.0 (4)	C74—C76—H76C	109.5
C13—C14—P1	104.8 (4)	H76A—C76—H76C	109.5
C16—C14—H14A	106.5	H76B—C76—H76C	109.5
C13—C14—H14A	106.5	C81—C77—C78	115.2 (5)
P1—C14—H14A	106.5	C81—C77—P32	115.0 (4)
C11—C15—H15A	109.5	C78—C77—P32	103.6 (4)
C11—C15—H15B	109.5	C81—C77—H77A	107.6
H15A—C15—H15B	109.5	C78—C77—H77A	107.6
C11—C15—H15C	109.5	P32—C77—H77A	107.6
H15A—C15—H15C	109.5	C79—C78—C77	107.0 (5)
H15B—C15—H15C	109.5	C79—C78—H78A	110.3
C14—C16—H16A	109.5	C77—C78—H78A	110.3
C14—C16—H16B	109.5	C79—C78—H78B	110.3
H16A—C16—H16B	109.5	C77—C78—H78B	110.3
C14—C16—H16C	109.5	H78A—C78—H78B	108.6
H16A—C16—H16C	109.5	C78—C79—C80	107.9 (5)
H16B—C16—H16C	109.5	C78—C79—H79A	110.1
C21—C17—C18	114.4 (4)	C80—C79—H79A	110.1
C21—C17—P2	114.9 (4)	C78—C79—H79B	110.1
C18—C17—P2	104.2 (4)	C80—C79—H79B	110.1
C21—C17—H17A	107.6	H79A—C79—H79B	108.4
C18—C17—H17A	107.6	C82—C80—C79	112.5 (5)
P2—C17—H17A	107.6	C82—C80—P32	115.8 (4)
C19—C18—C17	107.9 (5)	C79—C80—P32	104.7 (3)
C19—C18—H18A	110.1	C82—C80—H80A	107.8
C17—C18—H18A	110.1	C79—C80—H80A	107.8
C19—C18—H18B	110.1	P32—C80—H80A	107.8
C17—C18—H18B	110.1	C77—C81—H81A	109.5
H18A—C18—H18B	108.4	C77—C81—H81B	109.5
C18—C19—C20	108.3 (5)	H81A—C81—H81B	109.5
C18—C19—H19A	110.0	C77—C81—H81C	109.5
C20—C19—H19A	110.0	H81A—C81—H81C	109.5
C18—C19—H19B	110.0	H81B—C81—H81C	109.5
C20—C19—H19B	110.0	C80—C82—H82A	109.5
H19A—C19—H19B	108.4	C80—C82—H82B	109.5
C22—C20—C19	112.6 (5)	H82A—C82—H82B	109.5
C22—C20—P2	115.8 (3)	C80—C82—H82C	109.5
C19—C20—P2	105.3 (4)	H82A—C82—H82C	109.5
C22—C20—H20A	107.6	H82B—C82—H82C	109.5
C19—C20—H20A	107.6	F2—B1—F1	110.2 (5)
P2—C20—H20A	107.6	F2—B1—F4	110.4 (5)
C17—C21—H21A	109.5	F1—B1—F4	108.6 (5)
C17—C21—H21B	109.5	F2—B1—F3	110.3 (5)
H21A—C21—H21B	109.5	F1—B1—F3	108.7 (5)
C17—C21—H21C	109.5	F4—B1—F3	108.5 (5)
H21A—C21—H21C	109.5	F12—B3—F11	110.4 (6)
H21B—C21—H21C	109.5	F12—B3—F9	110.0 (6)
C20—C22—H22A	109.5	F11—B3—F9	109.1 (6)
C20—C22—H22B	109.5	F12—B3—F10	109.3 (6)
H22A—C22—H22B	109.5	F11—B3—F10	108.7 (6)
C20—C22—H22C	109.5	F9—B3—F10	109.3 (5)
H22A—C22—H22C	109.5	F7'—B2—F8'	126.5 (9)
H22B—C22—H22C	109.5	F7'—B2—F5	109.9 (9)
C32—C31—C38	128.2 (6)	F8—B2—F5	118.7 (7)
C32—C31—Rh2	73.3 (3)	F8'—B2—F5	122.9 (8)
C38—C31—Rh2	107.2 (4)	F7'—B2—F6'	116.9 (8)
C32—C31—H31A	113.3	F8'—B2—F6'	111.1 (8)
C38—C31—H31A	113.3	F8—B2—F7	110.5 (8)
Rh2—C31—H31A	113.3	F8'—B2—F7	123.4 (8)
C31—C32—C33	123.8 (6)	F5—B2—F7	108.6 (7)
C31—C32—Rh2	70.1 (3)	F6'—B2—F7	124.9 (7)
C33—C32—Rh2	109.1 (3)	F8—B2—F6	108.0 (7)
C31—C32—H32A	115.0	F5—B2—F6	107.9 (7)
C33—C32—H32A	115.0	F7—B2—F6	101.8 (5)
Rh2—C32—H32A	115.0	F7'—B2—F5'	101.2 (8)
C32—C33—C34	115.6 (5)	F8'—B2—F5'	97.8 (7)
C32—C33—H33A	108.4	F6'—B2—F5'	93.6 (6)
C34—C33—H33A	108.4		
